# Donor Size Doesn’t Impact *En Bloc* Kidney Transplant Outcomes: A Single-Center Experience and Review of Literature

**DOI:** 10.3389/ti.2022.10731

**Published:** 2022-10-12

**Authors:** Mario Spaggiari, Egor Petrochenkov, Hiteshi Patel, Pierpaolo Di Cocco, Jorge Almario-Alvarez, Alberto Fratti, Ivo Tzvetanov, Enrico Benedetti

**Affiliations:** ^1^ Department of Surgery, University of Illinois at Chicago, Chicago, IL, United States; ^2^ School of Medicine, University of Missouri-Kansas City, Kansas City, MO, United States

**Keywords:** kidney transplant, *en bloc* kidney, body weight, pediatric donor, review of literature

## Abstract

Few transplant programs use kidneys from donors with body weight (BW)<10 kg due to higher incidence of vascular and urological complications, and DGF. The purpose of this study was to investigate the non-inferiority of pediatric *en bloc* kidneys from donors with BW<10 kg. We performed a single-center retrospective analysis of *en bloc* kidney transplants from pediatric donor cohort (*n* = 46) from 2003 to 2021 and stratified the outcomes by donor BW (small group, donor BW<10 kg, *n* = 30; standard group, donor BW<10 kg, *n* = 16). Graft function, rate of early post-transplant complications, graft and patient survival were analyzed. Complication rates were similar between both groups with 1 case of arterial thrombosis in the smaller group. Overall graft and patient survival rates were similar between the small and the standard group (graft survival—90% vs. 100%, *p* = 0.09; patient survival—96.7 vs. 100%, *p* = 0.48). Serum creatinine at 1, 3, 5 years was no different between groups. Reoperation rate was higher in the small group (23.3% vs. 6.25%, *p* = 0.03). The allograft from small donors could be related to higher reoperation rate in the early post-transplant period, but not associated with lower long-term graft and patient survival.

## Graphical Abstract

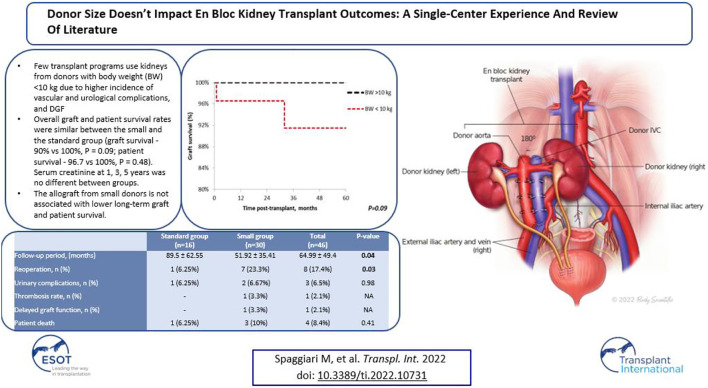

## Introduction

The United States kidney transplant waitlist has been constantly growing (1). In 2020, 37,408 new patients were added to the waitlist and 23,642 kidney transplants were performed (2). A 36.8% gap between patients who need the transplant and those who receive it forces transplant centers to look for new sources of donor organs.

Pediatric deceased donor *en bloc* kidneys (EBK) grafts are an underutilized source of suitable kidneys from transplant. Because of the perceived higher risk of technical complications, transplantation from *en bloc* kidneys is routinely performed only at a few transplant centers. A few reports showed higher incidence of vascular and urological complications (3,4), rejection, and delayed graft function with *en bloc* kidneys grafts (5). The risk of technical complications and poor graft survival is perceived to being associated with donor size.

We report on a single centre retrospective analysis on *en bloc* kidney transplants emphasizing outcomes and technical complications between the group of “small donors” (donor body weight (DBW≤10 kg) and the group of “standard” *en bloc* kidney donors (DBW>10 kg). A review of the literature has been performed for reference and comparison.

## Methods

### Study Population

This is a retrospective cohort analysis of *en bloc* kidney transplants in adult recipients, performed at an urban, academic institution between 2003 and 2021. Pediatric donors were stratified into 2 groups according to donor body weight (DBW): “standard group,” with DBW greater than 10 kg and “small group,” with DBW less than or equal to 10 kg. Donor demographics, including sex, race, age, weight, cause of death, cytomegalovirus (CMV) status, donation type (DBD/DCD) were obtained from United Network for Organ Sharing. This study was approved by IRB #2019-1320.

### Transplant

**FIGURE 1:**
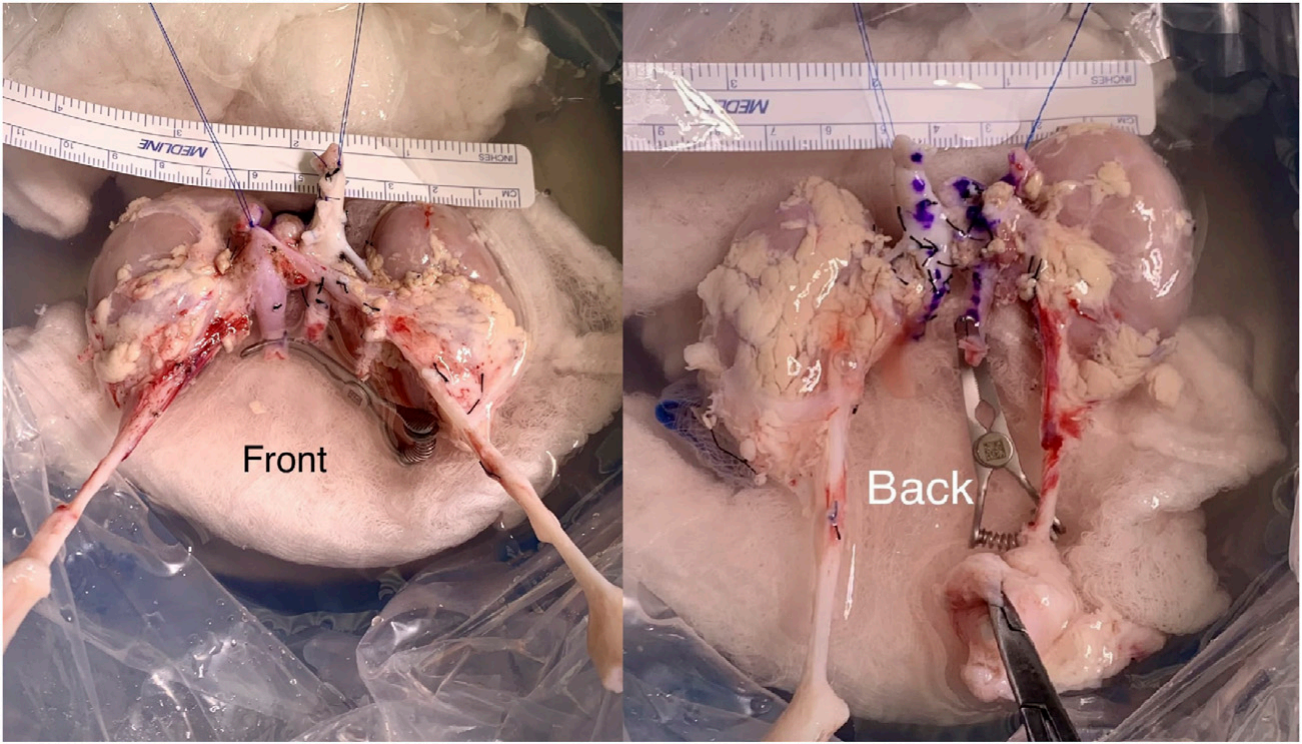
*En bloc* kidney graft during the backbench preparation stage.

**FIGURE 2:**
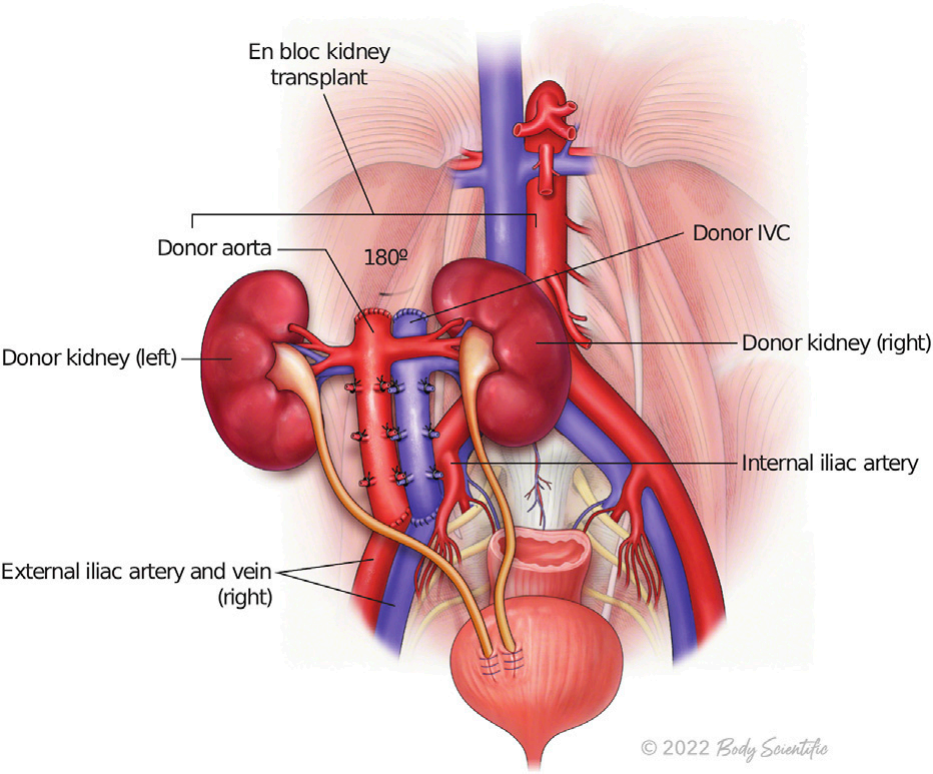
The illustration represents *en bloc* kidney transplant.

During backbench preparation of the graft, the proximal stump of the inferior vena cava and the aorta are oversewn with 6.0 Prolene. The distal ends of the IVC and the aorta are used for the anastomoses. If the bifurcation into iliac vein and iliac artery are present, they are used to create a wide patch. All aorta and IVC lumbars as well as adrenal and gonadal vessels are secured with 4/0 silk ties (Figure 1). Then, the graft is flipped 180° in order to align the aorta and IVC with recipient external iliac artery and vein respectively. End-to-side arterial anastomosis between the distal aorta of the graft and the external iliac artery are performed with 6.0 Prolene suture. The venous anastomosis is an end-to-side anastomosis between the distal IVC of the graft and the external iliac vein of the recipients sutured with 6.0 Prolene. Two separated ureteroneocystostomy anastomoses over double-J stents are routinely performed and sutured with 5.0 PDS (Figure 2).

Induction therapy consists of rabbit antithymocyte globulin and methylprednisolone followed by a rapid, 5-day steroid taper. Maintenance was achieved using mycophenolate and tacrolimus (8–12 ng/ml for the first 2 months, then 5–10 ng/ml thereafter). Institutional immunosuppression regimen did not change during the study period. All patients received antimycotic prophylaxis with fluconazole 200 mg during the first postoperative week. The antimicrobial prophylaxis included ampicillin/sulbactam and vancomycin. Cytomegalovirus prophylaxis was provided by valganciclovir 450 mg daily for 6 months except those with negative CMV serology in both donor and recipient. In that case, 1 month of acyclovir was used for herpes simplex virus prophylaxis. Anticoagulation prophylaxis consisted of aspirin-dipyridamole 25 mg/200 mg every 12 h for 2 months, followed by lifelong 81-mg aspirin daily.

### Outcomes

Cold Ischemia Time (CIT), Estimated Blood Loss (EBL) were analysed. Serum creatinine and eGFR values were collected at 6-months, 1-,3-,5-year follow up period. Delayed graft function (DGF) has been defined as the need of dialysis within the first week post-transplant. Rejection events, humoral (AMR), cellular (ACR), either empirically treated in case of sudden decrease of urine output associated with increase creatine or biopsy proven, have been reported. Post-transplant complications were collected: graft thrombosis, urinary leak, post-operative bleeding, and reoperation within the first 30 days.

### Survival

Patient and graft survival rates were estimated using Kaplan-Meier curves and compared between the groups using a log-rank test. Patients lost at the follow-up with functioning graft were included in this analysis.

### Statistical Analysis

Normally distributed continuous variables are expressed as mean ± standard deviation and non-normally distributed continuous variables as median (IQR). All continuous variables were normally distributed and reported as mean ± standard deviation and to compare between groups using analysis of variance test. Categorical variables were summarized as percentages and compared between groups using Fisher exact test. *p* values were calculated using 2-tailed tests and considered significant if less than 0.05. The statistical analysis was performed using IBM SPSS Statistics for Windows version 27 (IBM Corporation, Armonk, NY).

### Literature Review

PubMed database was searched using the terms “pediatric *en bloc* kidney,” “*en bloc* kidney,” and “transplantation.” We identified the studies published in the last 10 years, which included analysis of the EKT outcomes based on DBW or used DBW as the main criteria of the cohort stratification. The exclusion criteria from the literature research included the following: a cohort less than 10 patients; transplantation only to pediatric recipients, and transplantation of a single kidney. This yielded 6 articles which specifically detailed the outcomes of adult patients who received kidney grafts from pediatric donor (Table 1).

**TABLE 1:** Literature review: pediatric kidney transplant to adult recipients.

	Period	Number of patients	Results
Current study 2022	2003–2021	DBW>10 kg, *n* = 16	DGF—0%
			Rejection rate—12.5%
			5-y Graft survival—96.7%
			5-y Patient survival—100%
		DBW≤10 kg, n = 30	DGF—3.3%
			Rejection rate—10%
			5-y Graft survival—90%
			5-y Patient survival—100%
Peng et al. (6) 2021	2015–2019*	DBW≤5 kg, *n* = 32	DGF—34.4%
			Rejection rate - 12.5%
			5-y Graft survival—71.4%
			5-y Patient survival—96.9%
		5 kg<DBW≤20 kg, *n* = 143	DGF—23.1%
			Rejection rate—10.5%
			5-y Graft survival—89.5%
			5-y Patient survival—94.4%
		DBW>20 kg, *n* = 110	DGF—16.4%
			Rejection rate—10.9%
			5-y Graft survival—97.3%
			5-y Patient survival—99.1%
Lopez-Gonsalez et al. (7) 2022	1999–2021	*n* = 42, (mean DBW 11.3 ± 3.6 kg)	DGF—NR
			Rejection rate—NR
			Graft survival—83.3% (mean follow-up 73 months)
			5-y Patient survival - NR
Hafner-Giessauf et al(8) 2013	1990–2002	*n* = 13, (mean DBW 8 ± 3 kg)	DGF—NR
			Rejection - 7.7%
			5-y Graft survival—84.6%
			Patient survival—NR
Mitrou et al. (9) 2018	2000–2017**	DBW<10 kg, *n* = 11	DGF—45.5%
			Rejection rate—9%
			5-y Graft survival—81.8%
			5-y Patient survival—100%
		DBW>10 kg, *n* = 17	DGF—23.5%
			Rejection rate—5.8%
			5-y Graft survival—94.1%
			5-y Patient survival—82.4%
Troppmann et al. (10) 2018	2007–2015	DBW≤10 kg, *n* = 130	DGF—19.2%
			Rejection rate—NR
			5-y Graft survival—83.1%
			5-y Patient survival—93.5%
Choi et al. (11) 2017	1996–2016	*n* = 15, (mean DBW 13.14 kg)	DGF—20%
			Rejection rate—13%
			5-y Graft survival—92.9%
			5-y Patient survival—NR

n-number of patients; y-year; NR, not reported; DBW, donor body weight; DGF, delayed graft function; *—285 patients overall; **—28 patients overall.

## Results

### Study Population

Forty-six patients were identified for the analysis, 16 (34.78%) patients received the organ from donors with BW>10 kg (Range: 11.79–19.96) and 30 (65.22%) recipients had a donor with BW≤10 kg (Range: 3.18–9.98). Recipient baseline characteristics stratified by donor groups are presented in Table 2. The BMI of the recipients was significantly different between the groups (standard vs. small, 28.55 ± 6.88 kg vs. 24.39 ± 3.72 kg; *p* = 0.04). Fifteen (93.75%) out of 16 recipients in the standard group and 26 (86.7%) out 30 in the small group received dialysis pre-transplant. Duration of dialysis was not different between two groups (standard vs. small, 66.38 ± 36.83 vs. 50.63 ± 33.29; *p* = 0.16).

**TABLE 2:** Recipient characteristics stratified by DBW.

	Standard group (*n* = 16)	Small group (*n* = 30)	Total (*n* = 46)	*p*-value
Age, (years)	45.59 ± 14.42	48.41 ± 14.89	47.43 ± 14.63	0.54
Weight, (kg)	74.81 ± 18.49	67.82 ± 9.97	70.25 ± 13.76	0.18
BMI, (kg/m^2^)	28.55 ± 6.88	24.39 ± 3.72	25.84 ± 5.36	0.04
Sex, n (%)				0.99
• Male (%)	6 (37.5%)	17 (56.7%)	23 (50%)	
• Female (%)	10 (62.5%)	13 (43.3%)	23 (50%)	
Ethnicity, n (%)				0.1
• African-America	7 (43.75%)	13 (43.4%)	20 (43.47%)	
• Hispanic	8 (50%)	8 (26.6%)	16 (34.78%)	
• Caucasian	1 (6.25%)	3 (10%)	4 (8.6%)	
• Other	—	6 (20%)	6 (13.04%)	
CMV status, n (%)				0.4
• Positive	15	27	42	
• Negative	1	3	4	
Dialysis pretransplant, n (%)	15 (93.75%)	26 (86.7%)	41 (89.1%)	0.18
Duration of dialysis pretransplant, (month)	66.38 ± 36.83	50.63 ± 33.29	56.11 ± 34.99	0.16

n, number of cases; BMI, body mass index; CMV, cytomegalovirus.

Donors in the small group were younger (standard vs. small, 24.0 ± 13.91 vs. 4.5 ± 8.03; *p* = 0.00001). Despite the difference in BW between the groups, Δ Weight (Recipient-Donor) kg was not significantly different (*p* = 0.08). Male sex and African American ethnicity were dominant in both groups, with anoxia as the leading cause of death. Five DCD donors were in the cohort, 3 in the standard and 2 in the small group. Mean final serum creatinine was higher in smaller donors but without significant difference (0.38 ± 0.15 vs. 0.33 ± 0.2, *p* = 0.35). Pediatric kidney grafts were procured by the regional Organ Procurement Agency (Region 7) in 40 (86.9%) cases. Six kidneys were imported outside of the region (Ohio-3, Mississippi-1, Kentucky-1, Indianapolis-1), with 4 donors with BW≤10 kg. Donor characteristic summary is presented in Table 3.

**TABLE 3:** Donor characteristics stratified by DBW.

	Standard group (*n* = 16)	Small group (*n* = 30)	Total (*n* = 46)	*p*-value
Age, (months)	24.0 ± 13.91	4.5 ± 8.03	15.35 ± 14.4	0.00001
Weight, (kg)	15.14 ± 2.7	7.09 ± 2.15	9.89 ± 4.52	0.00000
Δ Weight (Recipient-Donor), (kg)	59.67 ± 18.27	60.73 ± 9.37	60.36 ± 12.9	0.83
Sex, n (%)				0.86
• Male (%)	11 (68.75%)	17 (56.7%)	28 (60.7%)	
• Female (%)	5 (31.25%)	13 (43.3%)	18 (39.3%)	
Ethnicity, n (%)				0.1
• African American	7 (43.75%)	16 (53.3%)	23 (50%)	
• Hispanic	2 (13%)	4 (13.3%)	6 (13.04%)	
• White	5 (31.25%)	10 (33.3%)	15 (32.6%)	
• Other	2 (13%)	—	2 (4.3%)	
Cause of death				NA
• Stroke	—	1	1	
• Anoxia	9	14	23	
• Head trauma	7	13	20	
• Other	—	2	2	
DCD/DBD	3/13	2/28	5/41	NA
CMV status, n (%)				0.6
• Positive	5 (31.25%)	7 (23.33%)	13 (28.26%)	
• Negative	11 (68.75%)	23 (76.67%)	34 (71.74%)	
Final serum creatinine, (mg/dl)	0.33 ± 0.2	0.38 ± 0.15	0.37 ± 0.17	0.35
Area of procurement, n (%)				0.16
• Region 7	12 (75.5%)	28 (93.3%)	40 (86.9%)	
• Outside of the Region	4 (25.5%)	2 (6.7%)	6 (13.1%)	

Region 7, Illinois, Wisconsin, South Dakota, North Dakota, Minnesota; n, number of cases; BMI, body mass index; CMV, cytomegalovirus; DCD, donation after cardiac death; DBD, donation after brain death.

### Outcomes

The mean follow-up in the standard group was significantly longer than in the small group (89.5 ± 62.55 vs. 51.92 ± 35.41 months; *p* = 0.04). No difference in intraoperative EBL was observed (*p* = 0.8). CIT was also similar between the standard and the small group, 13.8 ± 5.43 and 12.2 ± 5.7 h respectively (*p* = 0.36). The rate of reoperation within the first 30 days post-transplant was significantly higher in the group with DBW≤10 kg (6.25% vs. 23%; *p* = 0.03). Six (85%) out of 7 patients in the small group had a perinephric hematoma which required evacuation and additional hemostasis. No vascular thrombosis was observed in the standard group, while 1 out of 30 patients (3.3%) had arterial thrombosis in the small group. The thrombosis happened on POD 1 and led to graft loss. The rate of urological complications was not significantly different between the groups (standard vs. small, 6.25% vs. 67%; *p* = 0.98). Two patients in the small group had humoral rejection. Overall, 3 patients in the cohort experienced humoral rejection, and all cases were confirmed by biopsy and successfully treated with PLEX and IVIG. Additionally, two patients from the standard group had AMR, one of them experienced graft loss and was retransplanted. Only one (3.3%) episode of DGF was observed in the cohort, and the patient received the organ from a donor with BW≤10 kg. He recovered normal graft function after additional hemodialysis. All the outcomes and complications can be seen in Table 4.

**TABLE 4:** Outcomes and complications stratified by DBW.

	Standard group (*n* = 16)	Small group (*n* = 30)	Total (*n* = 46)	*p*-value
Cold ischemia time, (hours)	13.8 ± 5.43	12.2 ± 5.7	12.76 ± 5.65	0.36
Estimated blood loss, (ml)	136.56 ± 99.11	129.73 ± 93.10	131.63 ± 94.2	0.8
Follow-up period, (months)	89.5 ± 62.55	51.92 ± 35.41	64.99 ± 49.4	0.04
Reoperation, n (%)	1 (6.25%)	7 (23.3%)	8 (17.4%)	0.03
Urinary complications, n (%)	1 (6.25%)	2 (6.67%)	3 (6.5%)	0.98
Thrombosis rate, n (%)	—	1 (3.3%)	1 (2.1%)	NA
Rejection rate, n (%)	2 (12.5%)	3 (10%)	5 (10.9%)	0.58
Delayed graft function, n (%)	—	1 (3.3%)	1 (2.1%)	NA
Graft loss	—	3	3	0.58
Death with functioning graft	1	2	3	0.9
Patient death	1	3	4	0.41
Creatinine, (mg/dl)				
• 6 months	1.0 ± 0.23	1.45 ± 1.31	1.29 ± 1.08	0.09
• 1 year	0.94 ± 0.26	1.02 ± 0.35	0.99 ± 0.32	0.38
• 3 years	1.29 ± 1.66	1.60 ± 2.57	1.49 ± 2.26	0.69
• 5 years	1.26 ± 1.19	0.9 ± 0.36	1.05 ± 0.81	0.43
eGFR, (ml/min/1.73m^2^)				
• 6 months	81.07 ± 17.81	71.17 ± 30.45	74.54 ± 27.01	0.18
• 1 year	88.64 ± 22.23	88.16 ± 27.2	88.32 ± 25.35	0.95
• 3 years	93.68 ± 38.15	77.37 ± 35.97	83.19 ± 36.92	0.28
• 5 years	79.91 ± 30.63	93.86 ± 41.26	87.66 ± 36.25	0.42

n, number of cases; eGFR, estimated glomerular filtration rate.

**FIGURE 3:**
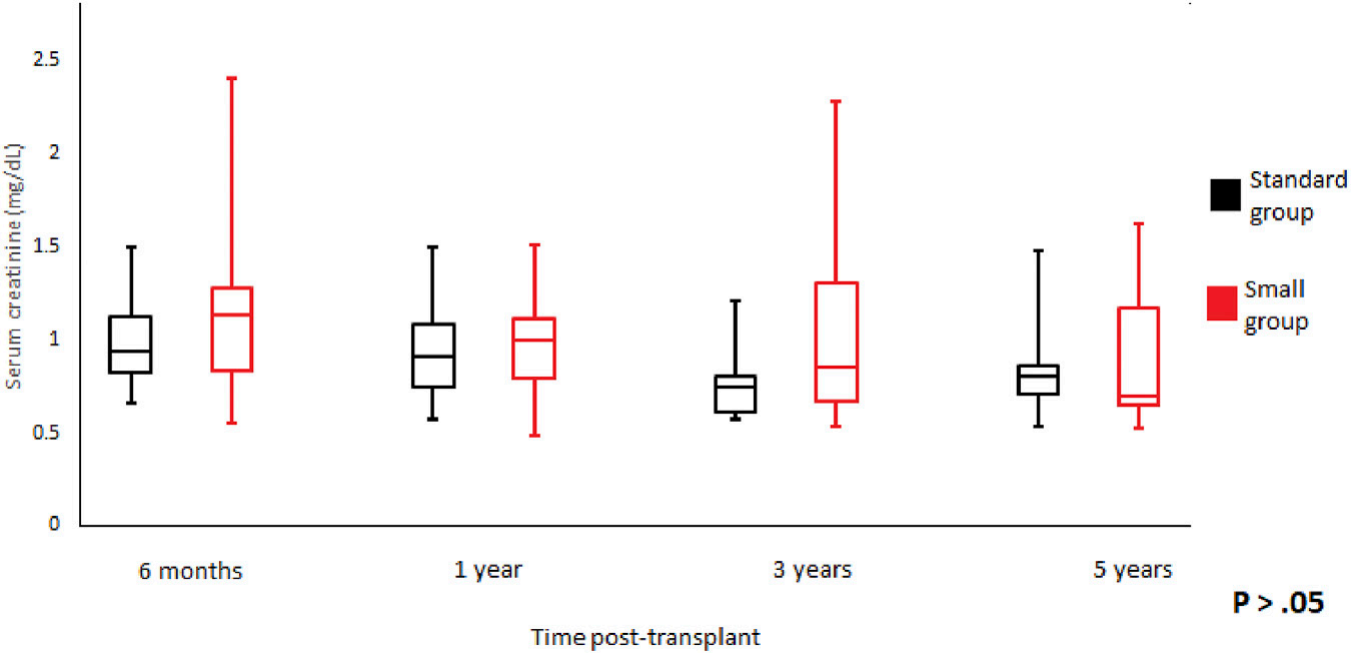
Serum creatinine trend according to donor weight during the study period. Mean serum creatinine and standard error of mean over scheduled time points. *p* > 0.05 at all time periods.

**FIGURE 4:**
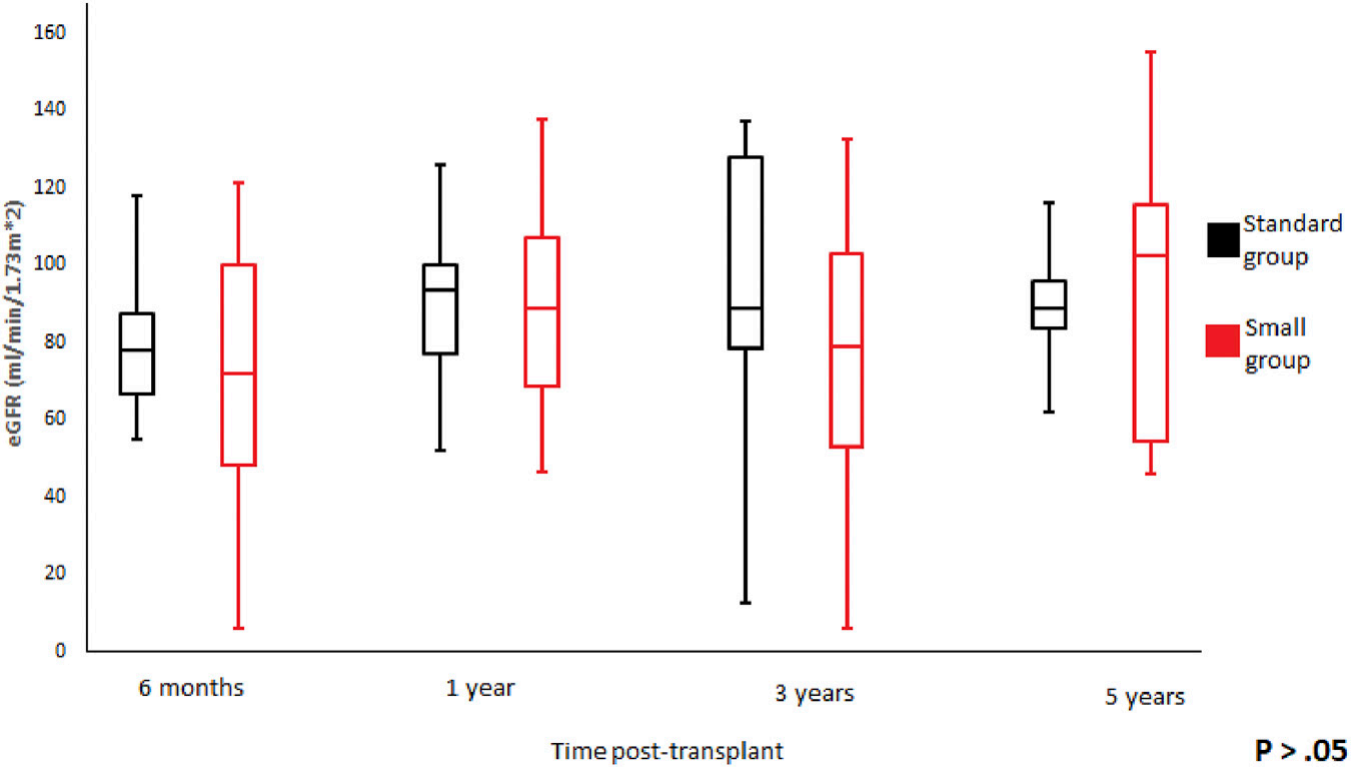
Glomerular filtration rate trend according to donor weight during the study period. Mean glomerular filtration rate and standard error of mean over scheduled time points. *p* > 0.05 at all time periods.

We did not observe any statistically significant differences in the graft function between the groups at 6-month, 1-, 3-, 5-year of follow-up (Figures 3, 4). Mean serum creatinine and eGFR levels in the standard group after 5 years post-transplant were 1.26 ± 1.19 mg/dl and 79.91 ± 30.63 ml/min/1.73 m^2^ respectively, and 0.9 ± 0.36 mg/dl and 93.86 ± 41.46 ml/min/1.73 m^2^ in the small group. Detailed graft function is presented in Table 4.

### Survival

**FIGURE 5:**
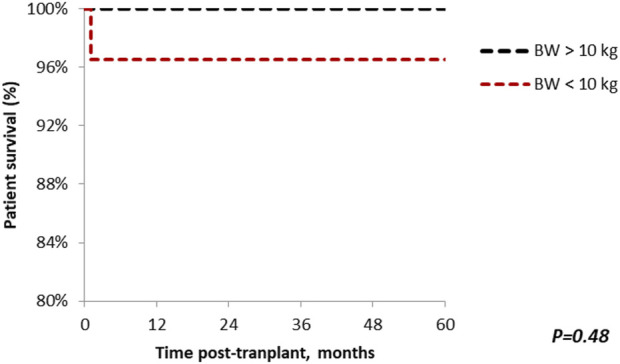
The Kaplan-Meier patient survival plot for *en bloc* kidney transplant patients. Patient survival in the standard and small groups at 1, 3, 5 years are 100% and 96.7% respectively. *p* > 0.05 was estimated using log-rank test.

**FIGURE 6:**
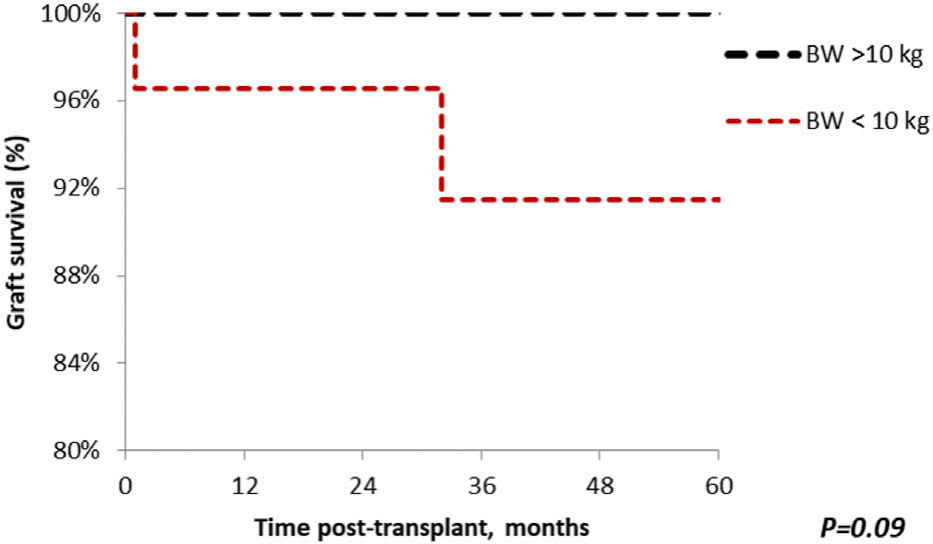
The Kaplan-Meier graft survival plot for *en bloc* kidney transplant patients. Graft survival in the standard group at 1, 3 and 5 years is 100%; the small group 96.7%, 90% and 90%. *p* > 0.05 was estimated using log-rank test.

Patient survival after 5 years was comparable among the groups (standard vs. small, 100% vs. 96.7%; *p* = 0.48), with median follow-up of 64.9 months (Range: 1–221) (Figure 5). Similar findings were observed in 5-year graft survival (standard vs. small, 100% vs. 90%; *p* = 0.09) (Figure 6). One graft was lost due to arterial thrombosis on POD1, one due to humoral rejection 32 months post-transplant in the setting of non-compliance, and the third one 11 years post-transplant. Three patient deaths were registered in the small group during 5-year follow-up; 2 of them occurred with functioning graft due to severe COVID-19 infection, and one patient had a myocardial infarction. The only deceased patient in the standard group passed due to COVID-19 infection. Three patients, all from the standard group, were lost in follow-up after 5, 4, and 4 years, respectively.

## Discussion

This study evaluates the outcomes of 46 pediatric *en bloc* kidney transplants using grafts from donors who weighed either greater or less than 10 kg. The primary outcome of this study is that renal function, graft and patient survival from donors with BW less than 10 kg are similar to such who received a pediatric transplant from donors with BW greater than 10 kg. We report excellent overall patient and graft survival rates for the cohort that included almost two-thirds of patients who received a kidney graft from extra small donors.

Recent publications have reported comparable graft survival between *en bloc* kidney transplant and both living and deceased donor adult kidney transplant (14, 13, 12). Suneja et al showed that the use of pediatric deceased donor kidneys has increased over the last few years but is still relative rare, especially from donors weighting <20 kg (13). Although it is a good source to expand the donor pool, almost 10% of kidneys from donors with BW≤20 kg are discarded (, 9, 14). A potential reason for that might be an extra degree of technical difficulties comparing to the grafts from adult donors, such as benching preparation of the organ or cystoureterostomy, so not every transplant center is comfortable with such procedures. As is reflected in our cohort, centers that do perform this procedure typically accumulate grafts from small donors from the different areas around them; almost 15% of the organs from this study were procured outside of the region and 25% outside of the state.

The largest number of EBK cases was reported by a group from China (6). Peng et al described 285 EBKs from 2015 to 2019 and showed how DBW affects the outcomes *via* a DBW<5 kg threshold. The authors demonstrated benefits for graft survival with increasing DBW by comparing groups with DBW<5 kg vs. 5 kg<DBW<20 kg vs. DBW>20 kg (71.4% vs. 89.5% vs. 97.3%; *p* <0.05). No difference in patient survival, rates of thrombosis, urological complications, and acute rejection. That is the only study to our knowledge that analysed this extra-small group of DBW<5 kg.

Study published by Mitrou et al. was similar to ours by design. It described 28 *en bloc* kidney transplants, including 11 cases with DWB<10 kg and with an overall graft and patient survival rate of 81.8% and 100% respectively, among this group (9).

In our institution we do not apply any exclusion criteria for recipients of *en bloc* kidney transplant. However, we try to allocate *en bloc* grafts to patients smaller than 80 kg regardless of BMI.

We are reporting only 1 (2.1%) graft thrombosis in this study. This rate is comparable with the rate mentioned by Bakir et al in an adult single kidney transplant series (16). The patient received the graft from a donor with a body weight of 4.99 kg. On POD1 he was reoperated due to decreased urine output and absence of any flow in the graft on Doppler US. Complete arterial and venous thrombosis of the graft vessels was founded, and graftectomy was performed. The patient was then successfully retransplanted.

In terms of surgical complications, we believe that it is important to highlight that we did not observe any significant difference in urinary tract complications between the two groups. Only two patients out of 46 were reoperated on POD5 and POD6 due to urinary leakage from one of the two reimplanted ureters. Additionally, one patient had postoperative stricture of the reimplanted ureter, which complicated with hydronephrosis and multiple UTIs. The overall rate of urinary complications in the cohort was 7.8%. This is on the low side of the range from recently published literature, which varies from 2.5 to 21% (15). Fananapazir et al, in a cohort of 225 EBKs, showed that DBW<10 kg is a significant risk factor for such complications (total *n* = 22 (9.8%); 12% vs. 2% for EBK donors <10 vs. ≥ 10 kg; *p* = 0.031). Stricture of the ureter was the most common complication (55%), followed by urinary leak (41%). But in 50% of cases these can be managed nonoperatively, and they do not affect graft and patient survival (17). In our series we did not perceive any difference, possibly due to a small number of cases. We prefer to perform two separate ureter anastomoses. Alternative techniques, with the utilization of the bladder cuff, have been described (18). However, since the vascular supply of the bladder patch cannot be properly assessed (with a higher risk of ischemia in male donors), we deem it safer to perform two separate anastomoses with partially shortened ureters. The final position of the graft, flipped 180°, allows for easier access to the pelvis in the case of urological complications.

Overall reoperation rate in the first 30 days post-transplant was significantly higher for patients in the small group. Besides when the graft was removed due to arterial thrombosis, six patients needed additional hemostasis and evacuation of a perinephric haematoma (without renewal of the vascular anastomosis). All of them received the graft from donors with BW <10 kg. One patient had multiple reoperations in the early post-transplant period (POD1—relaparotomy, evacuation of perinephric hematoma, POD6—reformation of the cystoureterostomy, POD15—enterolysis, and small bowel resection due to SBO). Despite the complicated early post-transplant period, after more than 5 years of follow-up the patient has maintained stable graft function. We explain the higher rate of perinephric hematomas in the small group by additional technical difficulty of performing the “ideal” benching of the organ: the submillimeter size of the lumbar branches, either venous or arterial, sometimes makes the recognition and ligation particularly challenging and increases the risk of post operative hematoma. All hematoma washouts happened within the first 2 days post-transplant.

In our cohort we had 5 DCD donors, three in the standard group (18.75%) and two from donors with BW<10 kg (6%). In these 5 cases, we are reporting 100% 5-year death-censored graft survival. Due to a limited number of this type of patients, we believe, that it is impossible to make any significant conclusions regarding the safe use of kidney from DCD donor with extra small body weight from our series. However, in previous literature, Troppmann at el demonstrated that DCD status impacts early post-transplant graft function but does not appear to impact added risk graft loss and long-term kidney function (10). Analysing 120 EBKs (65 DBD vs. 65 DCD) from donors with BW<10 kg they showed a higher, but not statistically significant, rate of DGF (25% vs. 14%), urological complications (15% vs. 12%), and graft loss (23% vs. 11%) in DCD group. DCD vs. DBD 5-year graft and patient survival were 87% vs. 91% and 90% vs. 97% respectively.

The results of our study should be interpreted after an acknowledgement of its limitations. The main limitation is the relatively small cohort size, yet this is one of the largest series of EBKs from donors with BW < 10 kg. In our knowledge, there are only two similar publications with bigger cohorts, both were mentioned previously (, 13, 6). However, there are also multiple studies in the literature with a smaller number of patients (21, 20, 19). With constantly improving surgical technique and post-transplant management, the lowest limit of DBW for kidney transplantation is not yet clear. Therefore, to maximize utilization and avoid discarding organs, we think that further investigation in a multicenter study on a larger cohort scale is necessary.

## Conclusion

To summarize, graft and patient survival rates after *en bloc* kidney transplantation from donors with BW<10 kg are not different from heavier donors. Renal function is unaffected by differences in DBW. The DBW<10 kg group is at an increased risk for surgical complications in early post-transplant period. This study provides evidence that kidney transplant from donors with BW less than 10 kg, with experience, is a potentially important method for expanding the pool of kidney donors.

## Data Availability

The raw data supporting the conclusion of this article will be made available by the authors, without undue reservation.

## Abbreviations

DBW, donor body weight; BW, body weight; CIT, cold ischemia time; EBL, estimated blood loss; CMV, cytomegalovirus; eGFR, estimated glomerular filtration rate; ACR, acute cellular rejection; AMR, antibody mediated rejection; BMI, body mass index; DBD, donation after brain death; DCD, donation after cardiac death; EBK, *en bloc* kidney; DGF, delayed graft function; IQR, interquartile range; POD, postoperative day; PLEX, plasma exchange; IVIG, intravenous immunoglobulin; IVC, inferior vena cava; n, number of cases.
