# Corrigendum: Donor Size Doesn’t Impact *En Bloc* Kidney Transplant Outcomes: A Single-Center Experience and Review of Literature

**DOI:** 10.3389/ti.2022.10982

**Published:** 2022-12-05

**Authors:** Mario Spaggiari, Egor Petrochenkov, Hiteshi Patel, Pierpaolo Di Cocco, Jorge Almario-Alvarez, Alberto Fratti, Ivo Tzvetanov, Enrico Benedetti

**Affiliations:** ^1^ Department of Surgery, University of Illinois at Chicago, Chicago, IL, United States; ^2^ School of Medicine, University of Missouri-Kansas City, Kansas City, MO, United States

**Keywords:** kidney transplant, *en bloc* kidney, body weight, pediatric donor, review of literature

In the original article, there was a mistake in the **Graphical Abstract** as published. The **Graphical Abstract** incorrectly contained the text of the full abstract. The corrected **Graphical Abstract** appears below.

**GRAPHICAL ABSTRACT:**
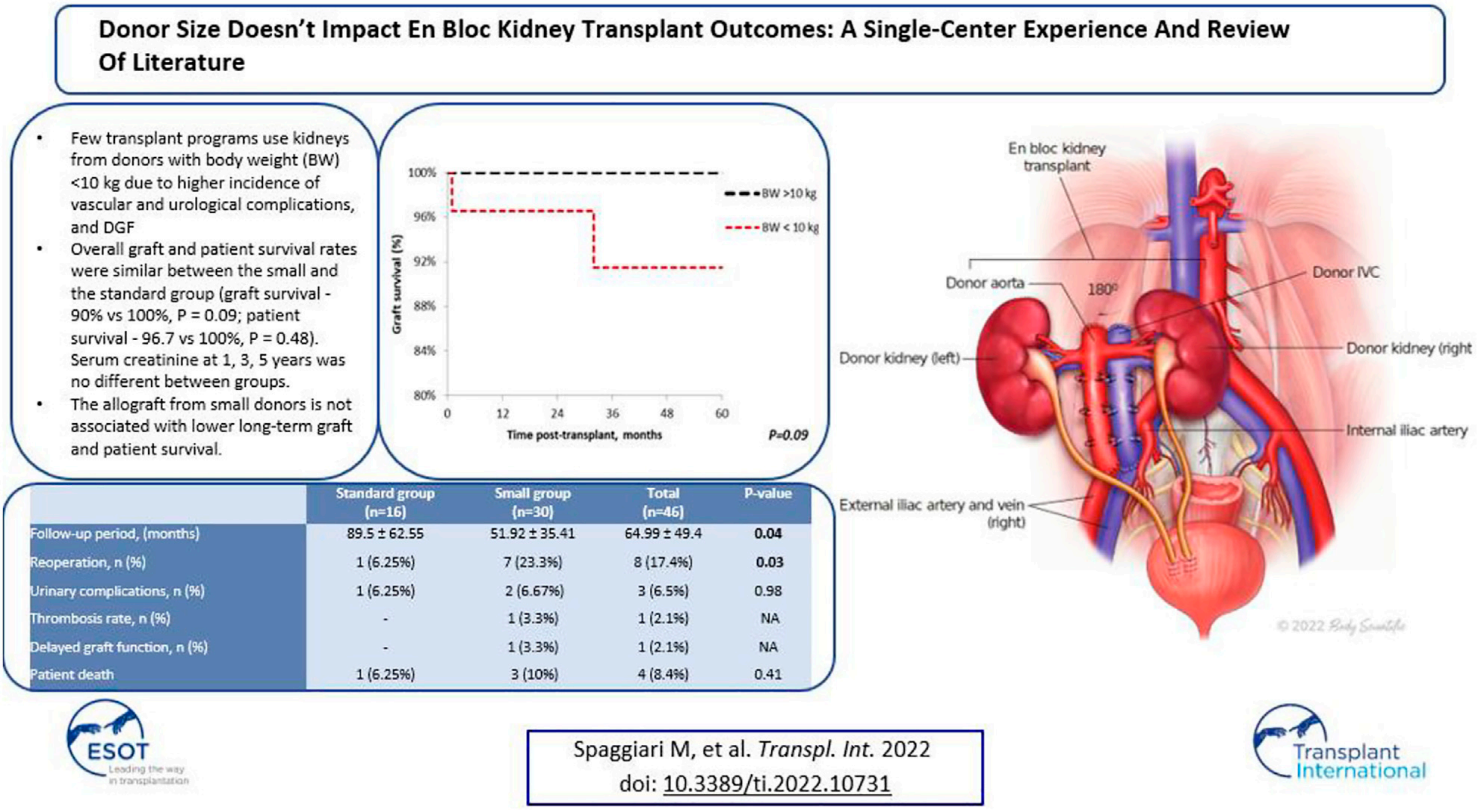


The authors apologize for this error and state that this does not change the scientific conclusions of the article in any way. The original article has been updated.

